# Psychological Distress in Urbanizing China: How Does Local Government Effectiveness Matter?

**DOI:** 10.3390/ijerph18042042

**Published:** 2021-02-19

**Authors:** Juan Chen, Lin Gong, Shenghua Xie

**Affiliations:** 1 Department of Applied Social Sciences, The Hong Kong Polytechnic University, Hong Kong, China; juan.chen@polyu.edu.hk (J.C.); lin1995.gong@connect.polyu.hk (L.G.); 2 School of Public Administration, Central China Normal University, Wuhan 430079, China

**Keywords:** psychological distress, worries, government effectiveness, urbanization, China

## Abstract

Despite growing literature identifying key individual, family, community, and environmental factors as causes for mental disorders during the process of urbanization, the role played by local government has not been taken into account. In this article, we investigate how the effectiveness of local government affects residents’ levels of psychological distress in areas of China undergoing urbanization. We measure the effectiveness of local governments according to their success in promoting access to the social security system through the distribution of social security cards among citizens. We hypothesize that higher local government effectiveness will reduce residents’ psychological distress by alleviating worries about medical expenses and elder care. Drawing on data from the 2018 Urbanization and Quality of Life Survey (*N* = 3229) conducted in 40 localities undergoing rural–urban transition, we estimate three-level mixed-effects regression models to test the research hypotheses, allowing random effects at the township/county and neighborhood levels while controlling for a series of individual attributes. The results demonstrate that local government effectiveness is negatively associated with residents’ psychological distress: effective local governments alleviate worries about medical expenses and elder care, and thereby reduce psychological distress. The findings indicate that, to reduce residents’ worries and psychological distress during the process of rural–urban transition, it is essential to improve local government effectiveness, particularly in promoting residents’ access to the social security system. Beyond demonstrating how local government effectiveness matters for residents’ psychological distress, our research also illustrates how to properly model locational parameters in analyses of individual well-being.

## 1. Introduction

The state of mental health has received growing attention as the level of urbanization continues to rise in developing countries. Studies have confirmed that psychological disorders are more common and complicated in urbanized areas [1]. While key individual, family, community, and environmental factors have been identified as causes for mental disorders during the process of urbanization [2], the role played by local government has not been taken into account. This is particularly important in China where both the scale and speed of urbanization have had an adverse effect on residents’ mental health [3,4], but the level of mental health literacy among the general population is relatively low, and people’s preconceptions about mental disorders and treatments need to be greatly improved [5,6]. In this study, we attempt to fill the research gap and address the following question: whether the effectiveness of local governments exacerbates or alleviates residents’ psychological distress in areas of China undergoing urbanization, and how.

We situate our research in the context of China’s implementation of the National New-Type Urbanization Plan (2014–2020). We measure local government effectiveness based on its success in promoting access to the social security system through the distribution of social security cards. We investigate the mental health status of residents of localities undergoing rural–urban transition and employ the Kessler Psychological Distress Scale (K10) as the outcome measure. The findings from our research enrich the existing literature and our understanding concerning government effectiveness and human well-being and provide insights regarding how local governments could improve their effectiveness in governance and reduce residents’ psychological distress.

We next present our research background and hypotheses. In the following sections, we introduce data from the 2018 Urbanization and Quality of Life Survey (*N* = 3229) before proceeding with the empirical analysis and results. We then discuss the study findings and their implications. We conclude the article with a highlight of the research contributions, the study limitations, and our suggestions for future research.

### 1.1. Government Effectiveness

Government effectiveness is one of the key indicators of good governance and a direct source of individual well-being [7,8,9]. According to the World Bank, government effectiveness can be broadly measured by “the quality of public services, the quality of the civil services and the degree of its independence from political pressures, the quality of policy formulation and implementation, and the credibility of the government’s commitment to such policies” [10]. Studies have examined the impact of government effectiveness on human welfare and well-being. Sacks and Levi, for instance, focus on the experience of citizens living in Africa, where famine remains a serious threat [11]. In their study, a government’s effectiveness is measured by its ability to facilitate reliable access to food for its citizens. If a government is effective, it will be able to deliver goods that individuals need to improve their social welfare. Government effectiveness is an important factor in combatting human trafficking [12]. In addition to the willingness to take action, a country must have the funds, institutional capacity, and human resources to implement an anti-trafficking policy. Government effectiveness also plays an important role in reducing child mortality, particularly in developing countries: those with more effective governments (i.e., competent bureaucracies and reliable public service delivery) are more efficient in providing healthcare services and, therefore, more likely to reach goals for health outcomes [13].

Some other studies focus on the links between government effectiveness and subjective well-being. Scholars argue that there are positive links between government effectiveness and the subjective well-being of citizens, and most empirical evidence supports this claim [8]. The quality of service delivery is generally considered more important than the degree of democracy in determining well-being, although in developed countries where service delivery has reached sufficient levels, the degree of democracy also shows positive links to subjective well-being [14,15]. Most studies of government effectiveness have been conducted at the national level: countries are viewed as consistent entities [16]. In recent years, scholars have attempted within-country comparisons, which demonstrate that cross-country analysis does not tell the whole story. There are many within-country variations that are, in part, the result of the effectiveness of lower levels of government [16,17]. Liu and colleagues, for instance, examine performance in public service delivery as one of the three key indicators of local government quality in China and find that it has the strongest positive impacts on urban respondents’ life satisfaction [15]. Survey-based measures of subjective well-being have been widely regarded as reliable indicators of human well-being in the literature on the effects of government [18]. The most widely used measure is a global cognitive assessment of happiness or life satisfaction [19]. Despite the growing literature on government effectiveness and subjective well-being, little research attention has been paid to measures of mental health, which is an important aspect of human well-being and is likely to be affected by government effectiveness as well.

### 1.2. Urbanization in China: Providing a Context

Over half of the world population now live in urban settings, and it is projected that five billion people will do so by 2030, and seven billion by 2050 [20]. Developed countries are already highly urbanized, so almost all of the urban expansion predicted will occur in developing countries [21]. A considerable body of evidence shows that having an effective local government is crucial in enabling efficacious urban planning, boosting development, and improving service provision and delivery, particularly in newly developed urban areas [22,23,24]. There is, therefore, an urgent need for scholars, policy-makers, urban planners, and service providers to understand the challenges associated with local governance, identify indicators of effective local government, and improve government effectiveness in localities undergoing rural–urban transition [25]. Following an agenda of territorial urbanization, the Chinese central government has established new cities, enlarged or merged existing ones, and eliminated others, in order to boost economic development and strengthen its power [26]. Between 1978 and 2010, the number of cities in China jumped from 193 to 658. As the cities proliferated, they also increased in size through extensive reclassification of previously rural land [27,28]. During this process, counties (and county-level cities) were converted to urban districts, rural townships (xiang) upgraded to towns (zhen), and towns and rural townships reclassified as street districts (jiedao) [29]. 

The current leadership in China continues to implement policies to promote urbanization. The Chinese household registration system (hukou), which was introduced in the 1950s to ensure economic and social control, consistently favors long-term residents in urban areas [30,31]. Without reforming the hukou and associated social insurance and welfare systems, further urbanization is likely to perpetuate unequal access to public services [32]. To address this disparity, in March 2014, the National New-Type Urbanization Plan (2014–2020) was launched, emphasizing people-centered urbanization and heralding a new era of urban development [33]. The Plan aims to increase the percentage of the population classified as urban to 60% by 2020, which would require relocating or reclassifying 100 million villagers. This goal was already achieved before the final months of 2020: 60% of Chinese citizens are now living in towns and cities. Moreover, the percentage is expected to rise to 70% by 2030 and 80% by 2050 [34]. In addition to increasing the urbanization rate, the Plan proposes to grant permanent and full urban status to 100 million new urbanites. It also aims to reduce rural–urban income and service disparities: the rural and urban social security systems will be integrated in order to narrow gaps in enrolment and access to services [35]. Despite these policy goals, scholars argue that the 2014 Plan was primarily designed to deal with the pressures of economic slowdown and to solve the problems created by past urbanization [35]. Local governments have been criticized for seeking financial gain through land sales rather than focusing on the general well-being of affected residents and communities [36]. For instance, effective local governments are expected to take responsibility for securing residents’ livelihoods by distributing social security cards and granting citizens easy access to the social security system. Yet the goal of making social security more equitable has not been realized: although enrolment reached 1.12 billion by March 2018 (representing 80.6% of the total population), huge variations still exist in access to the social security system [37]. 

The Chinese social security card is an all-in-one smart card that stores key personal information and provides the cardholder with access to a variety of government services. The card system relies on a central database that enables citizens to use the smart card to identify themselves, claim medical reimbursements, and make payments for health and other social services. The social security card system was piloted in Shanghai in 2001 and was created to address the operational constraints experienced by local government agencies and also fulfill the e-government directives of the central government. Access to the card was first limited to certain groups such as urban retirees and state employees [38], but since 2011, the social security card system has been expanded and implemented nationwide. Although the total number of cardholders keeps growing, there are striking disparities depending on locality [39]. 

In this study, we use the promotion of social security card distribution as a measure of local government effectiveness based on three rationales. First of all, nationwide social security card coverage is one of the main goals of the social security information system in China. Almost all local governments consider this issue at the top of their policy agenda and have initiated policies to address it [40]. Secondly, the social security card system provides a basis for standardized management, public service optimization, and rural–urban integration. The Shanghai municipal government, for example, was prompted by the introduction of the card to implement a city-wide information system to improve the service coordination among local administrative systems, including social security, public security, civil affairs, health insurance, and provident fund management [38]. The use of social security cards has improved the service quality and response speed of local governments [41]. Moreover, since September 2014, the e-certificate, which is based on the social security card system, has been established on a nationwide scale. The extended digital functions of the card include the ability to make inquiries and e-payments, and further boost e-government and the interactions between local governments and citizens [42]. 

### 1.3. Research Hypotheses

Although it does not focus specifically on psychological distress, research has established positive links between government effectiveness and citizens’ subjective evaluation of their life [8]. Effective government can lead to higher subjective well-being among residents, both directly and indirectly. Directly, people are better off living in a context of effective government; indirectly, effective government allows people to achieve higher levels of other metrics and reduce worries that threaten their overall well-being [8]. Most empirical evidence supports the positive links between government effectiveness in service delivery and subjective well-being [43,44]. Yet exactly how government effectiveness affects residents’ subjective well-being, and the magnitude of direct and indirect effects, have not been demonstrated, particularly in the context of lower levels of government in developing countries undergoing urbanization. 

In the case of China, scholars have expressed concern that the National New-Type Urbanization Plan downplays the challenges of regional heterogeneity and the effectiveness of local government [4,33]. Following the launch of the Plan, the National New Comprehensive Urbanization Pilot Program was implemented in 2014 [45]. The wide range of responses from the participating local governments revealed pressing concerns about the implementation and the effect of the Plan. Many local governments sought opportunities to obtain additional funding and land quotas for urban construction, and there were serious doubts as to whether these governments would make resident well-being their top priority [4,32]. Relatively few local governments appeared committed to promoting public service and social welfare during the process of rural–urban transition, so as to enhance residents’ mental health and reduce their psychological distress [42]. Based on these concerns and responses, we developed the main hypothesis:

**Hypothesis** **1.** *Local government effectiveness will have a direct effect on residents’ mental health: residents of localities with higher government effectiveness will report lower psychological distress.*

As noted, in addition to its direct effect, local government effectiveness may also affect residents’ mental health indirectly. During the process of urbanization, individuals are separated from their traditional agricultural communities. They are required to deal with a more complex, ever-changing urban society, and rely on their own efforts to make a living and pursue self-development [46]. In places where access to an urban social security system has not been secured, perceived risk and uncertainty about individual and family life are prevalent [3]. Scholars categorize worries as “micro-worries” and “macro-worries.” Micro-worries refer to potential problems of individuals and their circle, whereas macro-worries are associated with problems in broader social contexts, such as race/ethnicity, social class, nation state, or even the world [47]. Studies have documented the detrimental impact of micro-worries on psychological well-being, leading to higher levels of distress [48]. Macro-worries, on the other hand, are not associated with mental health [47]. Based on these theories and empirical evidence, we developed a second hypothesis:

**Hypothesis** **2.** *Local government effectiveness will have an indirect effect on residents’ mental health: localities with higher government effectiveness will alleviate residents’ micro-worries related to social security and thus reduce their psychological distress.*

## 2. Data, Measures, and Analytical Strategy

### 2.1. The 2018 Urbanization and Quality of Life Survey

The data for this study come from the Urbanization and Quality of Life Survey (*N* = 3229) we conducted in 2018. The survey targeted residents in 40 primary sampling units (PSUs), including 32 townships (street districts or towns) in newly urbanized areas and eight townships (towns or rural townships) considered to be potential sites of urbanization. Half of the PSUs were drawn from the list of the 2014 National New Urbanization Comprehensive Pilot Program [45]. The other 20 localities were selected from non-pilot areas using the Coarsened Exact Matching (CEM) technique [49]. The townships were located in different counties, county-level cities, and urban districts; the 40 PSUs covered 40 county-level administrative units. After the 40 PSUs were carefully sampled, we created a detailed geographical information system (GIS) that aggregated information at the arc-minute level within each PSU and generated spatial sample frames of physical areas [50].

Within each PSU, we randomly selected four half square minutes (HSMs) of latitude and longitude as secondary sampling units (SSUs)—about the size of a rural village or urban neighborhood. Within each SSU, we selected households for conducting the survey interviews. The target population was adults aged 18 to 75, regardless of their hukou status, who had resided in the township for at least six months and in the household for more than 30 days. Within each household, one eligible respondent was chosen using the Kish grid [50]. Ethical approval of research projects involving human subjects was obtained from the authors’ home institute. The survey fieldwork was carried out from April to June 2018. Face-to-face interviews were conducted using the computer-assisted personal interviewing (CAPI) system. After data checking and cleaning, we obtained the final valid sample of 3229 respondents with a response rate of 65.2%. Post-stratification weights were developed to adjust the survey respondents to the 2010 China Township Population Census Data on key demographic variables such as gender and migration status [29].

### 2.2. Measures

#### 2.2.1. Psychological Distress

We administered the Kessler Psychological Distress Scale (K10), a ten-item self-reported questionnaire that measures respondents’ psychological distress in the previous month. The validity of this measure has been tested in the Chinese context [51,52,53]. The Cronbach’s alpha was 0.93 for the study sample. We took the sum of the scores on the ten items. The final scores ranged from 10 to 50, with higher scores indicating higher levels of psychological distress.

#### 2.2.2. Individual-Level Variables

We included individual-level covariates in the analysis, including age, gender, marital status, education, occupation, party affiliation, household wealth, homeownership, hukou category, and migration status. We further controlled respondents’ chronic health conditions, enrolment in medical and pension insurance schemes, and possession of a local social security card. Respondents were asked whether they worried about paying large medical bills if they get seriously ill and about not having elder care in the future. The latter two measures were used as mediating variables. Table 1 shows details of the individual-level measures and their descriptive statistics.

#### 2.2.3. Government Effectiveness

We asked the respondents whether they possessed a local social security card at the time of interview. We aggregated the percentage of local social security cardholders at the PSU level and used it as the measure of local government effectiveness in the analysis. The percentage of cardholders in PSUs ranged from 1.25 to 97.53, with an average of 46.04 and a standard deviation (SD) of 29.43.

#### 2.2.4. Township/County-Level Covariates

To control the variations of local economic development, we collected gross domestic product (GDP) data at the county level from 2014 to 2017 (in 2014, the National New-Type Urbanization Plan was implemented, and 2017 was the year before the household survey was undertaken). We included the natural logarithm of GDP per capita in 2014 and the percentage of GDP growth from 2014 to 2017 in the analysis. 

To address the effects of survey sampling design, we also took into account whether the townships were in newly urbanized areas (vs. potential sites of urbanization) and whether the townships were in localities participating in the 2014 Pilot Program. 

Table 2 presents descriptive statistics and variable descriptions of the township/county-level measures.

### 2.3. Analytical Strategy

Because the data have a hierarchical structure with level-one units (individuals) nested in level-two units (SSUs), which, in turn, are nested in level-three units (PSUs), we estimated three-level mixed-effects models with random intercepts, allowing each PSU to have its own intercept and each SSU to have its own intercept relative to the PSU in which it was nested. Our dependent variable was K10, psychological distress. We used it as a continuous variable and estimated generalized linear regressions. To test the direct effect of local government effectiveness on residents’ psychological distress, we estimated six models. In Model 1, we only included individual-level covariates. In Model 2 and Model 3, we added the dichotomously coded individual social security cardholder and the percentage of social security cardholders in PSUs, respectively. Both variables were then included in Model 4. In Model 5, we controlled medical and pension insurance at the individual level. Finally, in Model 6, we added the township/county-level covariates. The regression results are reported in Table 3. 

To test the mediating effects of micro-worries, we further estimated logistic regression models first with “worry about medical expense” as the dependent variable and then with “worry about elder care” as the dependent variable. The individual- and township/county-level covariates remained the same as Model 6 in Table 3. We then added the two measures of worries to the model on psychological distress, first separately and then together. Table 4 presents results from the estimated regressions. Model 1 in Table 4 is identical to Model 6 in Table 3 but is included for ease of reference.

## 3. Results

### 3.1. Local Government Effectiveness and Psychological Distress

As shown in Table 3, the percentage of local social security cardholders in PSUs is negatively associated with individual psychological distress in Models 3 through 6, which indicates that the more effective the local government is in distributing the cards, the lower the psychological distress of the respondents. The coefficient slightly decreases from Model 4 to Model 5 after controlling for individual medical and pension insurance. At the individual level, cardholder status only shows a negative association with psychological distress in Model 2 and Model 4 before individual medical and pension insurance are controlled. 

Model 6 shows that, among township- and county-level covariates, the logarithm of county GDP per capita in 2014 is negatively associated with respondents’ psychological distress, which suggests that residents in localities with higher economic development reported better mental health status. However, the association between county GDP growth between 2014 and 2017 and individual psychological distress is very minor and not statistically significant.

At the individual level, enrolment in a pension scheme but not a medical insurance scheme appears to be associated with psychological distress. Chronic health conditions are strongly associated with psychological distress, age shows a reversed U-shaped association, and women reported higher distress. On the other hand, being married, higher education, household wealth, and homeownership are all associated with lower psychological distress.

### 3.2. Mediating Effects through Reducing Worries

As shown in Table 4, the percentage of local social security cardholders in PSUs is negatively associated with worry about medical expenses (standardized coefficient = −0.440, *p* < 0.05) in Model 2 and worry about elder care (standardized coefficient = 0.674, *p* < 0.01) in Model 3. Models 4 and 5 confirm that worry about medical expenses (standardized coefficient = 2.671, *p* < 0.001) and worry about elder care (standardized coefficient t = 2.308, *p* < 0.001) are strongly associated with psychological distress. 

We followed Baron and Kenny’s protocol to determine the existence and extent of a mediating effect on the relationship between local government effectiveness and individual psychological distress as a result of worries about medical expenses and elder care [54,55,56]. Adding worry about medical expenses in Model 4 reduces the standardized coefficient on the percentage of local social security cardholders from −0.719 (*p* < 0.01) to −0.563 (*p* < 0.05). The proportion of total effect that is mediated is 0.676. The Aroian version of the Sobel test yields z-value = 2.043 and *p*-value = 0.041. Similarly, adding worry about elder care in Model 5 reduces the standardized coefficient on the percentage of local social security cardholders from −0.719 (*p* < 0.01) to −0.500 (*p* < 0.05). The proportion of total effect that is mediated is 0.755. The Aroian version of the Sobel test further yields z-value = 2.669 and *p*-value = 0.008. These results indicate that the mediating effect is statistically significant, particularly in the case of worry about elder care. Finally, when both worry about medical expenses and worry about elder care are included in Model 6, the standardized coefficient on the percentage of local social security cardholders in PSUs further decreases to −0.475 (*p* < 0.05), while the standardized coefficients on other township/county and individual covariates remain stable.

### 3.3. Robustness Checks

To check the model specifications, we estimated three-level mixed-effects regressions with additional covariates. We included county public expenses per capita in 2014 and the growth of county public expense from 2014 to 2017 to take into account the fiscal capacity of local government. We used the number of medical beds per 10,000 residents in the county in 2014 as well as the percentage increase in medical beds in the county from 2014 to 2017 as measures of local service infrastructure. None of these additional covariates appeared to be significant in the estimated models, nor did the standardized coefficients on other variables change significantly. We therefore did not include these covariates in the models reported. The regression models and results are available upon request.

## 4. Discussion

Both our research hypotheses were confirmed with the empirical results presented in the previous section, which showed that respondents residing in localities with higher government effectiveness reported lower psychological distress and that a negative association between local government effectiveness and residents’ psychological distress was mediated by reducing micro-worries related to social security. Such findings indicate that, to reduce residents’ worries and psychological distress during the process of rural–urban transition, it is essential to improve local government effectiveness, particularly in promoting residents’ access to the social security system in the context of urbanizing China. The Chinese social security card system is a striking case in point, but huge variations exist across localities. Places where residents have a higher level of access to social services show better mental health outcomes. Yet, some localities may lack the resources necessary to implement the system themselves. This is particularly true when continuing urbanization will cause greater disparities in public service provision [32]. Financial and technological support should be allocated to these localities without enough resources so that local government effectiveness could be strengthened, and residents would have the same access to relevant services.

The findings also provide evidence that local government effectiveness in reducing residents’ psychological distress was mediated by alleviation of their worries about medical expenses and elder care. As China continues to implement the human-centered National New-Type Urbanization Plan, our research demonstrates that issues related to health and elder care are extremely important and should be a priority for both central and local governments. Moreover, according to our empirical results, the effect of worry about elder care is stronger and more significant than worry about medical expenses in meditating the relationship between local government effectiveness and residents’ psychological distress; in addition, respondents’ enrolment in a pension scheme but not a medical insurance scheme appears to be statistically associated with a lower level of psychological distress. We speculate that as China is experiencing a rapid, accelerating, and irreversible ageing of its population, the demand for elder care arrangement keeps growing. Although the growing concern about elder care is not the central focus of the present study, the issue deserves further research investigation and policy consideration.

## 5. Conclusions

Drawing on data from a national survey in 40 localities undergoing rural–urban transition in China, we investigated both the direct and indirect impact of local government effectiveness on residents’ psychological distress. Local government effectiveness was measured by the level of access to the social security system through the distribution of social security cards among citizens. We estimated three-level mixed-effects models to test our hypotheses. Although people can be more or less stressed and worried regardless of the type of problems they encounter [47], after controlling for individual attributes and allowing for random intercepts at neighborhood and township/county levels, our results still demonstrate that effective local governments significantly reduce residents’ psychological distress and their worries about medical expenses and elder care. 

The research enriches the existing literature concerning government effectiveness and human well-being. In particular, we confirm that mental health as an important aspect of human well-being is also closely related to the quality of local governance in the context of lower levels of government in developing countries undergoing urbanization. While demonstrating how local government effectiveness matters for residents’ psychological distress, our research further illustrates how to properly model locational parameters in analyses of individual well-being. Other than individual, family, community, and environmental factors, it is essential to take into account the role played by local government, which is an important source of individual well-being. 

As we conclude, two caveats in the analysis need to be noted. First, our research was conducted in the context of China’s implementation of the National New-type Urbanization Plan. To what extent our findings could have implications for other developing countries and regions requires additional and comparative analysis, although studies have documented that the process of urbanization creates huge challenges for local governance in many policies facing similar transitions and challenges [4,21]. Second, although we observed a direct association between worries and psychological distress, it is also likely that higher levels of psychological distress can worsen attitudes about life and amplify worries [48]. The relationship between worries and psychological distress requires further investigation to disentangle how worries may influence psychological distress and vice versa. 

## Figures and Tables

**Table 1:** Descriptive statistics of survey respondents and variable descriptions.

Variables	Means or Percentages	Variable Descriptions
K10 psychological distress (mean)	16.869 (0.163)	Kessler Psychological Distress Scale (K10): min = 10, max = 50
Social security cardholder (%)	46.231 (1.219)	Dichotomous: 1 = yes, 0 = no
Medical insurance (%)	91.680 (0.621)	Dichotomous: 1 = yes, 0 = no
Pension insurance (%)	53.775 (1.231)	Dichotomous: 1 = yes, 0 = no
Chronic health conditions (mean)	0.595 (0.020)	Number of pain-related, cardiovascular, respiratory, and other chronic disorders: min = 0, max = 6
Age (mean)	51.093 (0.440)	Years of age: min = 18, max = 75
Gender (%)	49.213 (1.209)	Dichotomous: 1 = female, 0 = male
Marital status (%)	79.123 (1.209)	Dichotomous: 1 = married, 0 = other
Education (mean)	7.073 (0.092)	Years of schooling: min = 0, max = 20
Occupation (%)	8.767 (0.741)	Dichotomous: 1 = professional/managerial, 0 = other
CCP membership (%)	6.726 (0.636)	Dichotomous: 1 = Chinese Communist Party (CCP) member, 0 = not a CCP member
Household wealth (mean)	2.364 (0.047)	An index based on ownership of a number of consumer items, such as an liquid-crystal-display television (LCD TV) and a car: min = 0, max = 7
Homeowner (%)	85.676 (1.461)	Dichotomous: 1 = homeowner, 0 = not a homeowner
Hukou (%)		Categorical:
Rural hukou	84.122 (0.756)	0 = rural hukou (reference)
Urban hukou	6.714 (0.522)	1 = urban hukou
Jumin hukou	9.165 (0.569)	2 = jumin hukou
Migration status (%)	16.708 (1.510)	Dichotomous: 1 = cross-town migrant, 0 = non-migrant
Worry about medical expenses (%)	69.692 (1.029)	Dichotomous: 1 = yes, 0 = no
Worry about elder care (%)	67.274 (1.018)	Dichotomous: 1 = yes, 0 = no

Notes: *N* = 3199. 30 cases with missing data were excluded. Data were weighted. Means or percentages are reported. Standard errors are in parentheses.

**Table 2:** Descriptive statistics of county/township-level variables.

Variables	Means or Percentages	Variable Descriptions
Percentage of social security cardholders in PSUs (mean)	46.039 (29.425)	Percentage of social security cardholders in primary sampling units (PSUs); min = 1.250, max = 97.531
County GDP per capita in 2014 (CNY, mean)	60,048 (36,946)	County gross domestic product (GDP) per capital in Chinese Yuan (CNY); min = 8998; max = 181,370
County GDP per capita in 2014 (ln, mean)	10.809 (0.659)	Natural logarithm of county GDP per capital; min = 9.105, max = 12.108
County GDP growth 2014–2017 (mean)	28.268 (9.324)	Percentage of county GDP growth from 2014 to 2017; min = 8.934, max = 46.274
Townships in newly urbanized areas (%)	80.000	Dichotomous; 1 = townships in newly urbanized areas, 0 = townships that are potential sites of urbanization
Townships in the 2014 Pilot Program (%)	50.000	Dichotomous; 1 = townships in the 2014 Pilot Program, 0 = townships not in the Pilot Program

Notes: *N* = 40. Means or percentages are reported. Standard deviations are in parentheses.

**Table 3:** Three-level mixed-effects models on psychological distress.

	Model 1	Model 2	Model 3	Model 4	Model 5	Model 6
**Township/county-level variables**						
Social security cardholders in PSUs			−1.119 ***	−0.885 ***	−0.727 **	−0.719 **
			(0.218)	(0.241)	(0.235)	(0.272)
County GDP per capita in 2014 (ln)						−0.523 **
						(0.184)
County GDP growth 2014–2017						−0.016
						(0.151)
Townships in newly urbanized areas						0.834
						(0.506)
Townships in the 2014 Pilot Program						0.072
						(0.446)
**Individual-level variables**						
Social security cardholder		−1.204 **		−0.887 *	−0.708	−0.711
		(0.399)		(0.413)	(0.435)	(0.435)
Medical insurance					−0.797	−0.769
					(0.716)	(0.711)
Pension insurance					−1.045 *	−1.045 *
					(0.472)	(0.470)
Chronic health conditions	1.999 ***	2.016 ***	2.033 ***	2.040 ***	2.048 ***	2.042 ***
	(0.173)	(0.167)	(0.172)	(0.168)	(0.169)	(0.171)
Age	2.059	2.148	1.975	2.064	2.475*	2.571*
	(1.149)	(1.170)	(1.157)	(1.174)	(1.172)	(1.173)
Age (squared)	−2.666 *	−2.725 *	−2.598 *	−2.656 *	−2.948 **	−2.998 **
	(1.061)	(1.072)	(1.069)	(1.077)	(1.078)	(1.078)
Female	0.580*	0.550*	0.587*	0.566*	0.554*	0.574*
	(0.272)	(0.273)	(0.269)	(0.270)	(0.269)	(0.271)
Married	−1.185 **	−1.216 **	−1.210 **	−1.225 **	−1.185 **	−1.192 **
	(0.413)	(0.411)	(0.413)	(0.411)	(0.415)	(0.422)
Years of schooling	−0.664 **	−0.603 **	−0.635 **	−0.594 **	−0.543 *	−0.502 *
	(0.210)	(0.217)	(0.210)	(0.216)	(0.215)	(0.209)
Professional/managerial occupation	0.286	0.277	0.298	0.285	0.315	0.301
	(0.576)	(0.578)	(0.570)	(0.572)	(0.570)	(0.574)
Chinese Communist Party (CCP) member	−0.597	−0.492	−0.542	−0.476	−0.424	−0.440
	(0.587)	(0.596)	(0.579)	(0.588)	(0.595)	(0.605)
Household wealth	−1.189 ***	−1.143 ***	−1.216 ***	−1.168 ***	−1.130 ***	−1.131 ***
	(0.179)	(0.179)	(0.172)	(0.171)	(0.171)	(0.171)
Homeowner	−2.193 **	−2.099 **	−2.209 **	−2.134 **	−2.105 **	−2.142 **
	(0.754)	(0.764)	(0.730)	(0.743)	(0.735)	(0.736)
Hukou (ref.: Rural hukou)						
Urban hukou	0.114	0.349	0.138	0.290	0.460	0.442
	(0.486)	(0.490)	(0.494)	(0.491)	(0.498)	(0.500)
Jumin hukou	−1.117 **	−0.971 *	−0.942 *	−0.883 *	−0.810 *	−0.784
	(0.421)	(0.420)	(0.395)	(0.406)	(0.390)	(0.405)
Cross-town migrants	−1.076	−1.194	−1.049	−1.147	−1.242 *	−1.157
	(0.605)	(0.634)	(0.612)	(0.636)	(0.617)	(0.616)
Constants	19.750 ***	20.244 ***	19.762 ***	20.123 ***	21.276 ***	20.560 ***
	(0.799)	(0.807)	(0.728)	(0.748)	(0.932)	(1.044)
**Random-effects parameters**						
Variance (county/township)	2.033***	1.457**	0.743	0.789	0.596	0.242
	(0.613)	(0.556)	(0.408)	(0.424)	(0.394)	(0.373)
Variance (neighborhood | county/township)	2.884 **	2.997 **	2.869 **	2.950 **	2.910 **	2.914 **
	(0.991)	(1.023)	(0.985)	(1.006)	(1.008)	(1.006)
**Intraclass Correlation Coefficient (ICC)**						
County/township	0.040	0.029	0.015	0.016	0.012	0.005
Neighborhood | county/township	0.094	0.088	0.072	0.075	0.071	0.064
**Observations**						
Number of county/townships	40	40	40	40	40	40
Number of neighborhoods	159	159	159	159	159	159
Number of respondents	3199	3199	3199	3199	3199	3199
**Log pseudo likelihood**	−10,682.531	−10,674.916	−10,673.175	−10,669.256	−10,661.232	−10,657.244

Note: Data were weighted. Standardized coefficients are reported. Robust standard errors are in parentheses. * *p* < 0.05, ** *p* < 0.01, *** *p* < 0.001.

**Table 4:** Three-level mixed-effects models on psychological distress and worries.

	Model 1	Model 2	Model 3	Model 4	Model 5	Model 6
	Psychological Distress	Worry about Medical Expenses	Worry about Elder Care	Psychological Distress	Psychological Distress	Psychological Distress
**Township/county-level variables**						
Social security cardholders in PSUs	−0.719 **	−0.440 *	−0.674 **	−0.563 *	−0.500 *	−0.475 *
	(0.272)	(0.204)	(0.230)	(0.243)	(0.243)	(0.238)
County GDP per capita in 2014 (ln)	−0.523 **	−0.159	−0.144	−0.466 *	−0.465 *	−0.447 *
	(0.184)	(0.158)	(0.182)	(0.192)	(0.184)	(0.191)
County GDP growth 2014–2017	−0.016	0.338*	0.393	−0.150	−0.138	−0.188
	(0.151)	(0.171)	(0.223)	(0.154)	(0.141)	(0.152)
Townships in newly urbanized areas	0.834	−0.691	−0.675	1.085 *	1.022 *	1.134 *
	(0.506)	(0.470)	(0.579)	(0.519)	(0.503)	(0.520)
Townships in the 2014 Pilot Program	0.072	0.149	0.227	0.014	−0.061	−0.047
	(0.446)	(0.381)	(0.443)	(0.445)	(0.435)	(0.445)
**Individual-level variables**						
Worry about medical expenses				2.671 ***		2.035 ***
				(0.381)		(0.449)
Worry about elder care					2.308 ***	1.319 ***
					(0.338)	(0.400)
Social security cardholder	−0.711	−0.335 *	−0.413 **	−0.597	−0.561	−0.541
	(0.435)	(0.145)	(0.148)	(0.423)	(0.426)	(0.423)
Medical insurance	−0.769	0.131	−0.126	−0.824	−0.725	−0.782
	(0.711)	(0.237)	(0.197)	(0.686)	(0.710)	(0.690)
Pension insurance	−1.045*	0.120	−0.010	−1.086 *	−1.022 *	−1.063 *
	(0.470)	(0.154)	(0.143)	(0.450)	(0.455)	(0.446)
Chronic health conditions	2.042 ***	0.325 ***	0.383 ***	1.906 ***	1.899 ***	1.856 ***
	(0.171)	(0.050)	(0.053)	(0.175)	(0.170)	(0.174)
Age	2.571 *	1.885 ***	2.002 ***	1.760	1.814	1.525
	(1.173)	(0.331)	(0.511)	(1.243)	(1.225)	(1.257)
Age (squared)	−2.998 **	−2.154 ***	−2.271 ***	−2.066	−2.145	−1.804
	(1.078)	(0.315)	(0.495)	(1.136)	(1.124)	(1.152)
Female	0.574 *	0.173	−0.063	0.514	0.604 *	0.545 *
	(0.271)	(0.107)	(0.092)	(0.269)	(0.268)	(0.268)
Married	−1.192 **	0.097	−0.164	−1.230 **	−1.134 **	−1.186 **
	(0.422)	(0.157)	(0.160)	(0.406)	(0.414)	(0.404)
Years of schooling	−0.502 *	0.055	−0.011	−0.526 *	−0.511 *	−0.525 *
	(0.209)	(0.063)	(0.071)	(0.215)	(0.208)	(0.213)
Professional/managerial occupation	0.301	−0.295	−0.187	0.494	0.411	0.509
	(0.574)	(0.170)	(0.225)	(0.552)	(0.555)	(0.545)
CCP member	−0.440	−0.376	−0.076	−0.233	−0.393	−0.257
	(0.605)	(0.235)	(0.250)	(0.568)	(0.587)	(0.565)
Household wealth	−1.131 ***	−0.370 ***	−0.338 ***	−0.958 ***	−0.996 ***	−0.919 ***
	(0.171)	(0.066)	(0.066)	(0.168)	(0.168)	(0.169)
Homeowner	−2.142 **	−0.379	−0.439	−1.984 **	−1.992 **	−1.934 **
	(0.736)	(0.319)	(0.339)	(0.678)	(0.737)	(0.693)
Hukou (ref.: Rural hukou)						
Urban hukou	0.442	0.159	−0.134	0.347	0.439	0.366
	(0.500)	(0.271)	(0.180)	(0.434)	(0.505)	(0.449)
Jumin hukou	−0.784	−0.431 *	−0.229	−0.580	−0.707	−0.590
	(0.405)	(0.213)	(0.255)	(0.402)	(0.374)	(0.393)
Cross-town migrants	−1.157	−0.436	0.124	−1.012	−1.255 *	−1.104
	(0.616)	(0.275)	(0.240)	(0.594)	(0.596)	(0.586)
Constants	20,560 ***	1.997 **	2.535 **	18,411 ***	18,615 ***	17,806 ***
	(1.044)	(0.681)	(0.779)	(0.981)	(1.031)	(0.996)
**Random-effects parameters**						
Variance (county/township)	0.242	0.732*	1.096**	0.401	0.224	0.390
	(0.373)	(0.301)	(0.383)	(0.427)	(0.433)	(0.458)
Variance (neighborhood | county/township)	2.914 **	0.430 *	0.231 *	2.582 **	2.811 **	2.619 **
	(1.006)	(0.167)	(0.096)	(0.900)	(1.003)	(0.920)
**ICC**						
County/township	0.005	0.164	0.237	0.008	0.005	0.008
Neighborhood | county/township	0.064	0.261	0.287	0.062	0.063	0.063
**Observations**						
Number of county/townships	40	40	40	40	40	40
Number of neighborhoods	159	159	159	159	159	159
Number of respondents	3199	3199	3199	3199	3199	3199
**Log pseudo likelihood**	−10,657.244	−1581.6751	−1540.9434	−10,616.83	−10,627.357	−10,609.250

Note: Data were weighted. Standardized coefficients are reported. Robust standard errors are in parentheses. * *p* < 0.05, ** *p* < 0.01, *** *p* < 0.001.

## Data Availability

The data presented in this study can be available upon request.
